# Management of bone health in patients with cancer: a survey of specialist nurses

**DOI:** 10.1007/s00520-019-04858-2

**Published:** 2019-06-15

**Authors:** Lawrence Drudge-Coates, Erik van Muilekom, Julio C de la Torre-Montero, Kay Leonard, Marsha van Oostwaard, Daniela Niepel, Bente Thoft Jensen

**Affiliations:** 1 Department of Urology, King’s College Hospital NHS Foundation Trust, Denmark Hill, London, SE5 9RS UK; 2 Antoni van Leeuwenhoek, Netherlands Cancer Institute, Amsterdam, Netherlands; 3 San Juan de Dios School of Nursing and Physical Therapy, Comillas Pontifical University, Madrid, Spain; 4 St Luke’s Radiation Oncology Centre at St James’s Hospital, Dublin, Ireland; 5 Máxima Medisch Centrum, Eindhoven/Veldhoven, Netherlands; 6 Amgen (Europe) GmbH, Rotkreuz, Switzerland; 7 Aarhus University Hospital and Centre of Research in Rehabilitation, Aarhus University, Aarhus, Denmark

**Keywords:** Bone, Cancer, Neoplasm metastasis, Nursing education, Bone drug effects

## Abstract

**Background:**

Patients with cancer can experience bone metastases and/or cancer treatment–induced bone loss (CTIBL), and the resulting bone complications place burdens on patients and healthcare provision. Management of bone complications is becoming increasingly important as cancer survival rates improve. Advances in specialist oncology nursing practice benefit patients through better management of their bone health, which may improve quality of life and survival.

**Methods:**

An anonymised online quantitative survey asked specialist oncology nurses about factors affecting their provision of support in the management of bone metastases and CTIBL.

**Results:**

Of 283 participants, most stated that they worked in Europe, and 69.3% had at least 8 years of experience in oncology. The most common areas of specialisation were medical oncology, breast cancer and/or palliative care (20.8–50.9%). Awareness of bone loss prevention measures varied (from 34.3% for alcohol intake to 77.4% for adequate calcium intake), and awareness of hip fracture risk factors varied (from 28.6% for rheumatoid arthritis to 74.6% for age > 65 years). Approximately one-third reported a high level of confidence in managing bone metastases (39.9%) and CTIBL (33.2%). International or institution guidelines were used by approximately 50% of participants. Common barriers to better specialist care and treatment were reported to be lack of training, funding, knowledge or professional development.

**Conclusion:**

This work is the first quantitative analysis of reports from specialist oncology nurses about the management of bone metastases and CTIBL. It indicates the need for new nursing education initiatives with a focus on bone health management.

**Electronic supplementary material:**

The online version of this article (10.1007/s00520-019-04858-2) contains supplementary material, which is available to authorized users.

## Background

Up to three-quarters of patients with advanced prostate or breast cancer develop bone metastases, and bone metastases are also common in patients with other solid tumours (e.g. lung and kidney cancers) [11–13]. Bone lesions are present in approximately 90% of patients with multiple myeloma [14]. Bone metastases and bone lesions are associated with life-altering morbidity due to skeletal-related events (SREs), such as pathologic fractures, the need for radiotherapy or surgery to the bone, and spinal cord compression [13, 21, 26, 31], and with reduced overall survival [13, 18, 34]. There is a negative association between SREs and survival (e.g. in men with metastatic castration-resistant prostate cancer: mortality hazard ratio 1.67; 95% confidence interval 1.22–2.30; *p* = 0.001) [19]. Bone is also affected by many cancer treatments, which can induce bone loss (cancer treatment–induced bone loss [CTIBL]). CTIBL is associated with increased fracture risk and increased SRE burden in patients with advanced cancer [12, 13]. Therefore, bone health needs to be maintained not only in patients with bone metastases but also in those at risk of CTIBL. With continuing improvements in cancer survival, the management of bone metastases and CTIBL is becoming increasingly important to improve outcomes [13] and to reduce the burden on patients and healthcare provision.

Bisphosphonates and the monoclonal antibody denosumab help to maintain bone mineral density by targeting osteoclast activity, thereby reducing the rate of bone resorption [7]. As the main agents used for the prevention of SREs in cancer, denosumab and zoledronic acid are used for the prevention of SREs in adults with advanced malignancies involving bone [3, 24]. Both agents are also used at a lower dose and frequency for the treatment of bone loss in osteoporosis [4, 25]. Lower dose denosumab is also indicated in the cancer setting for treatment of bone loss associated with hormone ablation in men with prostate cancer at increased risk of fractures [4]. This indication also includes treatment of women with breast cancer receiving an aromatase inhibitor [16]. Detailed guidance on the use of zoledronic acid or denosumab is available in international and country-specific guidelines (e.g. those of the European Society for Medical Oncology [ESMO] [13], the National Institute for Health and Care Excellence [NICE] [23], Cancer Care Ontario [2] and National Comprehensive Cancer Network [18]). The ESMO guidelines for bone health in patients with cancer recommend the use of a bisphosphonate or denosumab in patients with metastatic bone disease which should commence at diagnosis and continue indefinitely throughout the course of the disease. More specifically, zoledronic acid or denosumab should be given to patients with bone metastases from breast cancer as well as castration-resistant prostate cancer and bone metastases, whether they have bone-specific symptoms or not. Patients with bone metastases from advanced lung cancer, renal cancer and other solid tumours are advised to take zoledronic acid or denosumab if they have a life expectancy of over 3 months and are considered at high risk of SREs [13].

As with any intervention, these agents are associated with some adverse events. Although infrequent, osteonecrosis of the jaw (ONJ) can be of particular concern [3, 24]. The incidence of ONJ in patients receiving bisphosphonates for bone metastases (*n* = 252) was 1.5%, 4.3% and 7.7% in those treated for 4–12 months, 13–24 months and 25–48 months, respectively [6]. In long-term phase 3 extension studies of denosumab, the cumulative patient-year adjusted incidence of ONJ in patients with breast cancer was 2.5%, with exposure for a median of 19.1 months, and in patients with prostate cancer was 2.8%, with exposure for a median of 12.0 months [28]. However, most cases occurred in patients with a history of tooth extraction, poor oral hygiene and/or use of a dental appliance [6, 28]. Therefore, when considering the use of bisphosphonates or denosumab, the clinician should include a dental examination and preventive dentistry, if necessary, in assessing the risk–benefit ratio for the patient.

In addition to bisphosphonate or denosumab therapy, bone health can be further maintained through calcium and vitamin D dietary supplementation, as well as lifestyle changes such as smoking cessation, reduction in alcohol consumption and increased participation in weight-bearing exercise. Regular bone mineral density screening of patients who are at risk of bone loss can also inform management of bone health [13].

Considering the indications/contraindications for bisphosphonates and denosumab and the associated adverse events, as well as the non-drug recommendations to maintain bone health, treatment decisions for patients with bone metastases depend on a variety of factors. Therefore, bone health should be managed using a multidisciplinary approach, to include not only oncologists, orthopaedic surgeons, specialist nurses and interventional radiologists but also specialists in palliative medicine and symptom control with expertise in bone complications from cancer [13, 20].

Although practices vary across the globe, specialist nurses are most often registered nurses who are clinical experts in a specialty area. Roles undertaken by specialist oncology nurses are highly variable and include, but are not limited to, coordination of services, patient advocacy, physical examinations and diagnostics, provision of treatments and patient education [9, 17, 30]. There is growing evidence from patient surveys that patients benefit from support provided by specialist oncology nurses in terms of disease-related problems, quality of life, continuity of care and unmet needs [5, 10, 29]. In particular, education on the importance of adhering to bisphosphonate or denosumab therapy can greatly improve persistence and compliance and, subsequently, patient outcomes [9, 17]. Nurses are in a key position to inform and to educate patients and their families about their disease and bone health because of their increased contact time with patients (often due to lengthy treatment administrations in nurse-led clinics [32]). They are also ideally placed to collaborate with other members of the multidisciplinary team, such as oncologists, pharmacists and physiotherapists, to coordinate the provision of optimal patient care. However, to our knowledge, there are no quantitative analyses of reports from specialist oncology nurses about the management of bone metastases and CTIBL. The aim of this survey was to gain information about these nurses’ awareness and knowledge of, and involvement in, managing bone metastases and CTIBL. Nurse confidence in managing bone health and perceived barriers to care for patients with bone metastases and CTIBL are also reported.

## Methods

### Online survey design and participants

This study gathered information from specialist oncology nurses using an anonymised online survey (www.surveymonkey.co.uk) designed, tested and launched by a steering committee of specialist oncology nurses (Online Resource 1). The survey comprised 39 questions about work setting, nursing experience and awareness of bone metastases, bisphosphonates and denosumab (referred to as bone-targeted agents in the questionnaire) and CTIBL. The URL to the survey was distributed to specialist nurses via the European Association of Urology Nurses (EAUN) website and via email to nursing societies, most of which were based in Europe (EAUN, Irish Association for Nurses in Oncology [IANO], Irish Association of Urology Nurses [IAUN], Spanish Association of Urology Nursing [ENFURO], Spanish Oncology Nursing Society [SEEO], Spanish Society of Palliative Care [SECPAL], Australia and New Zealand Urological Nursing Society [ANSUNS]). The survey was also advertised at the European Oncology Nursing Society 2016 Congress. The survey was available in English, French, German, Hebrew, Italian and Spanish and was conducted from 12 November 2014 to 16 January 2017. The English version of the questionnaire is available in Online Resource 2. Participants were not incentivised or remunerated for their contribution.

### Data analyses

So that all responses were captured, the analyses include participants who answered at least one question (total sample). All analyses are descriptive, and the results are presented as number and percentage. All percentages are calculated using the total number of included participants.

## Results

This study includes the responses for the questionnaires in English, French, German and Spanish (*n* = 150, 27, 83 and 23, respectively); the Italian and Hebrew questionnaires elicited very few responses (*n* = 2 and 1, respectively). In total, responses were available from 283 nurses working in specialist fields. A total of 244 (86.2%) participants answered the question regarding the geographical location of their institution. Among responding participants, nurses were from 17 countries, including Switzerland, Ireland, Spain, the UK and Australia (Table 1).

**Table 1:** Demographics, specialties and roles of survey participants

Category	Responses (*N* = 283), *n* (%)
Geographic location of institute	
Switzerland	108 (38.2)
Ireland	51 (18.0)
Spain	29 (10.3)
UK	20 (7.1)
Australia	13 (4.6)
Other^1^	23 (8.1)
Did not answer	39 (13.8)
Oncology experience (years)	
< 1	12 (4.2)
1 to < 4	23 (8.1)
4 to < 8 years	47 (16.6)
8 to < 12 years	54 (19.1)
12 to < 15 years	41 (14.5)
≥ 15 years	101 (35.7)
Did not answer	5 (1.8)
Workplace setting	
Outpatient clinics	155 (54.8)
Inpatient wards	132 (46.6)
Community care	12 (4.2)
Other	42 (14.8)
Did not answer	15 (5.3)
Institution type	
Public	90 (31.8)
General hospital	78 (27.6)
Comprehensive cancer centre	39 (13.8)
Private	32 (11.3)
Mixed	18 (6.4)
Other	15 (5.3)
Did not answer	11 (3.9)
Specialist area (multiple answers possible)	
Medical oncology	144 (50.9)
Breast cancer	67 (23.7)
Palliative care	59 (20.8)
Urology	57 (20.1)
Radiotherapy	36 (12.7)
Paediatric care	18 (6.4)
Cancer treatment–induced bone loss	17 (6.0)
Surgery	16 (5.7)
Geriatric care	10 (3.5)
Bone health	10 (3.5)
Orthopaedics	3 (1.1)
Other	30 (10.6)
No specialisation	17 (6.0)
Did not answer	4 (1.4)
Patient care roles (multiple answers possible)	
Providing information for patients on the adverse events of treatment	144 (50.9)
Providing psychosocial support for patients	135 (47.7)
Administering treatment/diagnostic interventions	111 (39.2)
Education of nurses	111 (39.2)
Patient advocacy	108 (38.2)
Pain management/palliative care	95 (33.6)
Providing information for patients on treatment options	84 (29.7)
Monitoring disease progression	81 (28.6)
Prescription of medication	34 (12.0)
Undertaking diagnostic tests (e.g. biopsy, imaging [e.g. DXA scan])	25 (8.8)
Did not answer	115 (40.6)

*DXA*, dual energy X-ray absorptiometry

Austria (*n* = 1), Azerbaijan (*n* = 1), Croatia (*n* = 1), Cyprus (*n* = 1), Denmark (*n* = 1), France (*n* = 1), Greece (*n* = 2), Italy (*n* = 1), Malta (*n* = 2), Netherlands (*n* = 10), Portugal (*n* = 1), Turkey (*n* = 1)

### Demographics of participants

Overall, over two-thirds of participants (69.3%) had at least 8 years of experience in oncology (Table 1). Nearly all participants worked in outpatient clinics or inpatient wards, and a fifth was also involved in community care and other settings (Table 1). There was a wide range of institution types represented in the survey (e.g. public sector, general hospital) (Table 1). Overall, the most common areas of specialisation were medical oncology, breast cancer, palliative care, urology and/or radiotherapy; some participants stated that they specialised in bone health or CTIBL (Table 1). The most commonly reported roles in patient care were providing information on the adverse events of treatment to improve bone health (50.9%) and providing psychosocial support for patients (47.7%); a variety of other roles were reported, while 40.6% of participants did not answer the question (Table 1).

### Knowledge and involvement of specialist nurses in the management of bone health

**Fig. 1:**
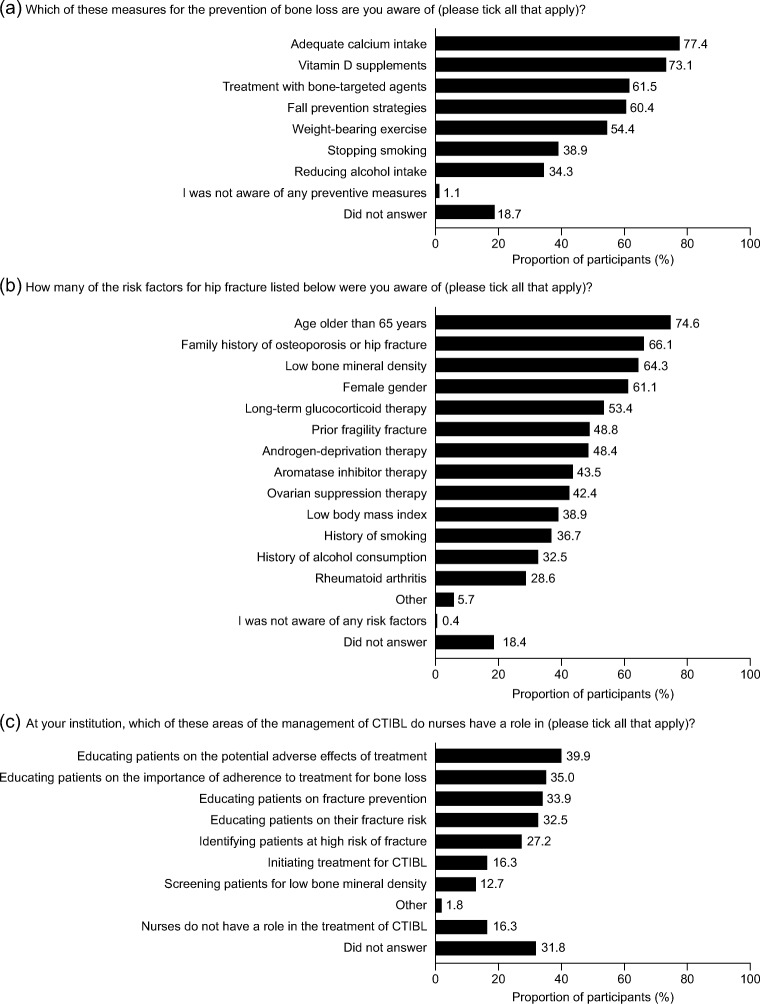
Participants’ awareness of and involvement in the management of bone health (*N* = 283). *CTIBL*, cancer treatment–induced bone loss

Knowledge of the various measures for bone loss prevention and risk factors for hip fracture was variable. For bone loss prevention, 77.4% of nurses were aware that adequate calcium intake is a protective measure, but only 34.3% knew that reducing alcohol intake is also a bone-protective measure. Similarly, although 74.6% knew that being aged over 65 years is a risk factor for hip fracture, only 28.6% knew that rheumatoid arthritis is also a risk factor. Few were not aware of any of the preventive measures or risk factors (1.1% and 0.4%, respectively), and approximately 18% did not answer the questions (Fig. 1a, b). Regarding involvement in the management of CTIBL, 12.7–39.9% stated one or more specific roles, principally in patient education; 16.3% stated that nurses do not have a role in managing CTIBL at their institution, and 31.8% did not answer (Fig. 1c).

**Fig. 2:**
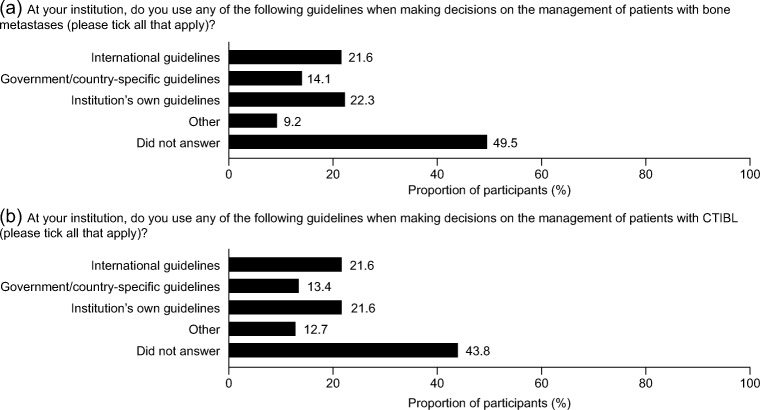
Participants’ use of guidelines for the treatment of **a** patients with bone metastases and **b** patients with CTIBL (*N* = 283). *CTIBL,* cancer treatment–induced bone loss

Approximately one-half of participants acknowledged using guidelines for both the management of bone metastases and CTIBL; 49.5% and 43.8%, respectively, did not answer (Fig. 2a, b). The most frequently used guidelines for the management of bone metastases were international guidelines (21.6%) and institution’s own guidelines (22.3%). Similar figures were found for CTIBL (Fig. 2a, b). A similar pattern was obtained for awareness of these guidelines (data not shown).

### Confidence and comprehension of specialist nurses in managing bone health

**Fig. 3:**
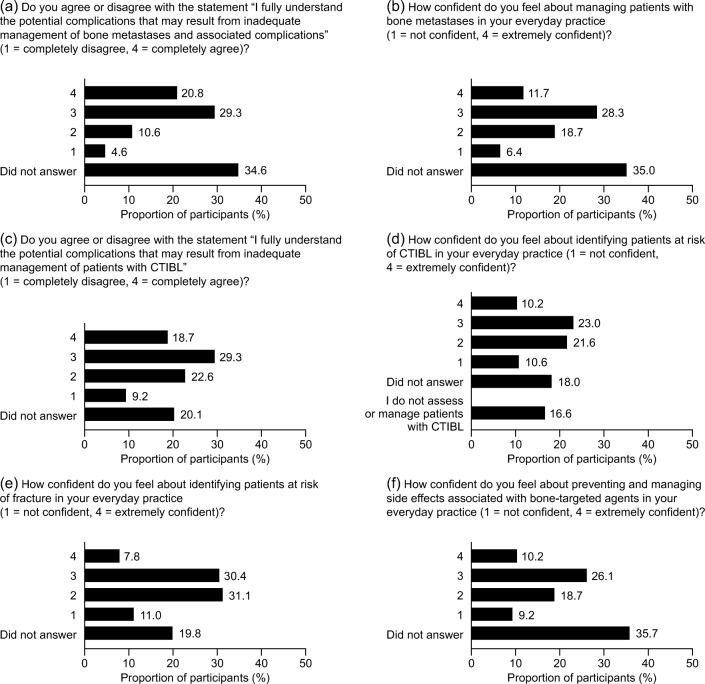
Confidence and comprehension in managing bone health (*N* = 283). Total values may not equal 100.0% due to rounding off. *CTIBL*, cancer treatment–induced bone loss

When asked about their understanding of potential complications that may result from inadequate management of bone metastases, approximately half of the participants (50.1%) stated that they agreed or completely agreed that they fully understood; 34.6% did not answer (Fig. 3a). However, when it came to their confidence in managing patients with bone metastases (rating their confidence from 1 = not confident to 4 = extremely confident), only 40.0% of participants reported a high level of confidence (level 3 or 4), compared with 25.1% reporting a low level of confidence (level 1 or 2) and 35.0% provided no answer (Fig. 3b). The comprehension and confidence in nurses with regard to CTIBL were broadly similar to those for bone metastases: 48.0% agreed or completely agreed that they fully understood the potential complications that may result from inadequate management of CTIBL; 20.1% did not answer (Fig. 3c). However, only 33.2% of participants reported a high level of confidence (level 3 or 4) in identifying patients at risk of CTIBL; 32.2% reported a low level of confidence (level 1 or 2); 16.6% stated that they do not assess or manage patients with CTIBL and 18.0% provided no response (Fig. 3d). In respect of identifying patients at risk of fracture, 42.1% reported a low level of confidence (level 1 or 2), with only 38.2% reporting a high level of confidence (level 3 or 4) and 19.8% did not respond (Fig. 3e). In terms of preventing and managing side effects associated with bisphosphonates and denosumab, 36.3% reported a high level of confidence (level 3 or 4); 27.9% reported a low level of confidence (level 1 or 2), and 35.7% did not answer (Fig. 3f).

### Perceived barriers to better management of patients with bone metastases

**Fig. 4:**
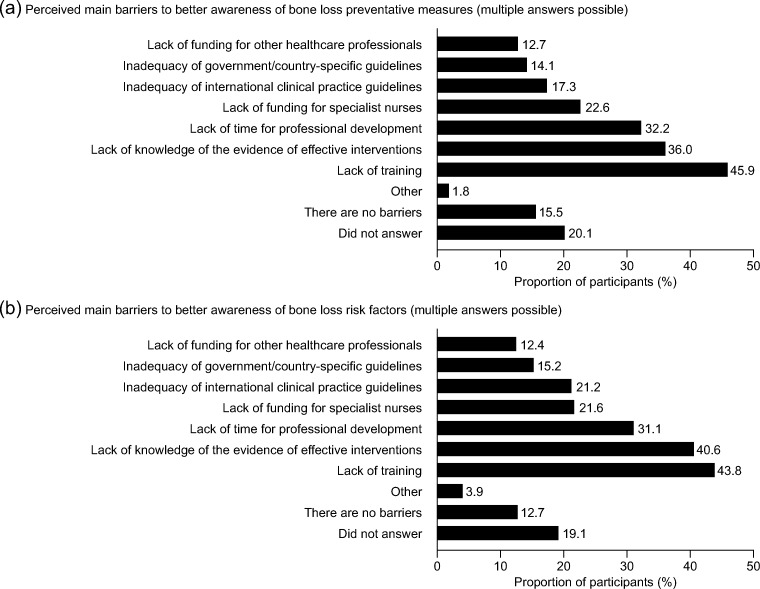
Barriers to better awareness of preventive measures of and risk factors for bone loss (*N* = 283)

**Fig. 5:**
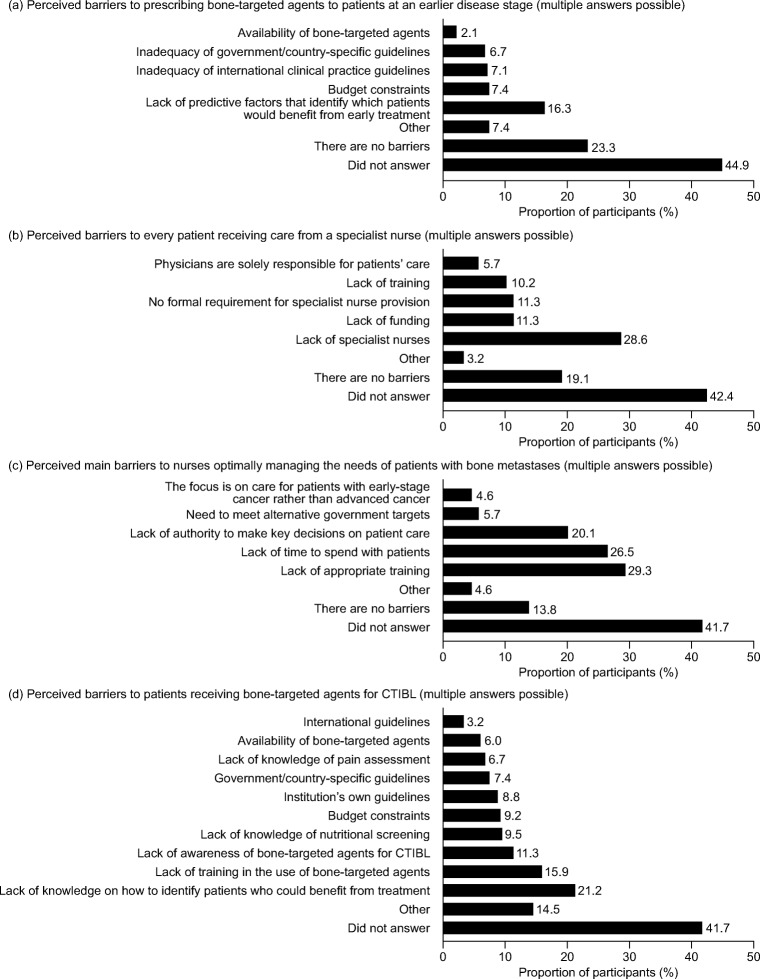
Barriers to specialist care and treatment with a bone-targeted agent related to bone metastases (**a**–**c**) and CTIBL (**d**) (*N* = 283). *CTIBL*, cancer treatment–induced bone loss

Common barriers to better awareness of preventive measures and risk factors for bone loss related to lack of training, lack of knowledge, lack of time for professional development and lack of funding for specialist nurses; 20.1% and 19.1%, respectively, did not answer (Fig. 4a, b). For prevention of SREs, the most common reasons for initiation of treatment with bisphosphonates or denosumab at the participants’ institutions were diagnosis of bone metastases (40.9%), an SRE (25.6%), castration-resistant prostate cancer (22.8%) and advanced cancer (20.3%); 2.1% reported not using bisphosphonates or denosumab for the prevention of SREs. The most common barriers to patients receiving bisphosphonates or denosumab at an earlier disease stage were lack of predictive factors that identified which patients would benefit from early treatment, budget constraints, inadequacy of international or government/country-specific clinical guidelines and availability of bisphosphonates and denosumab; 23.3% stated that there were no barriers, and 44.9% did not respond (Fig. 5a). The most common barriers to every patient receiving care from a specialist nurse were lack of specialist nurses, lack of funding, no formal requirement for specialist nurse provision, lack of training and physicians being solely responsible for patients’ care; 19.1% reported that there were no barriers, and 42.4% did not answer (Fig. 5b). The most common barriers to nurses optimally managing the needs of patients with bone metastases were lack of appropriate training, lack of time to spend with patients, lack of authority to make key decisions on patient care, a need to meet alternative government targets and the focus being on care for patients with early-stage cancer rather than advanced cancer; 13.8% reported that there were no barriers, and 41.7% did not answer the question (Fig. 5c).

### Perceived barriers to better management of patients with CTIBL

The most common barriers to patients receiving bisphosphonates or denosumab for CTIBL were lack of knowledge on how to identify patients who could benefit from treatment, lack of training in the use of bisphosphonates and denosumab and lack of awareness of bisphosphonates and denosumab for CTIBL; 41.7% did not answer (Fig. 5d). This question generated the highest proportion of participants selecting the option of ‘Other’ (14.5%) compared with the other questions about knowledge and treatment barriers (3.2–7.4%) (Fig. 5).

For the treatment of CTIBL, the most common reasons for initiation of bisphosphonates or denosumab at the participants’ institutions were the identification of multiple risk factors for fracture and prescription of an aromatase inhibitor, ovarian suppression or androgen-deprivation therapy. The use of T-scores less than − 2.0 without or with additional risk factors was less frequently reported; 4.6% reported not using bisphosphonates or denosumab for CTIBL.

### Summary of results

In summary, over 250 nurses from 17 different countries and with experience in over 11 different specialisations responded to this survey. Knowledge of measures for bone loss prevention and risk factors for hip fracture were variable, with some factors more commonly recognised than others. While approximately half of the participants reported that they understood the potential complications of inadequate management of bone metastases and CTIBL, fewer reported high levels of confidence in treating patients with these conditions. Several barriers to patients receiving the best possible care were reported, including lack of training for nurses, lack of specialist nurses, lack of time to spend with patients and lack of knowledge.

## Discussion

### Management of bone health by specialist nurses

To our knowledge, the present survey is the first to investigate quantitative data of specialist oncology nurses’ reports about factors that affect their provision of support in the management of bone metastases and CTIBL. The findings suggest that there is room for improvement in the levels of awareness, knowledge and involvement of nurses in managing bone metastases and CTIBL in patients with cancer. Only two preventive measures for bone loss (adequate calcium intake and vitamin D supplements) were recognised by approximately three-quarters of participants; other measures, including treatment with bisphosphonates or denosumab, were known by no more than two-thirds of participants. Less than half of participants gave a response that was in line with the ESMO 2014 guidelines to prevent SREs in patients with cancer and at risk of CTIBL (i.e. that bisphosphonates or denosumab should be initiated when bone metastases are diagnosed) [13]. Similarly, some risk factors for hip fractures in CTIBL (e.g. previous fragility fracture and androgen-deprivation therapy) [3, 13, 24] were identified by less than half of the participants. As the most commonly reported roles of the participants in the management of CTIBL were related to patient education, including adverse effects, the importance of treatment adherence, fracture prevention and fracture risk, it is particularly important that nurses have a high level of awareness of bone health management.

### Awareness, confidence and barriers to treatment

Approximately half of the participants stated that they understood the potential complications of poor management of bone metastases and CTIBL; approximately 15–30% reported low awareness, and 20–35% did not respond. Additionally, only 25–40% of participants were confident in treating patients with bone metastases, identifying those at risk of CTIBL and fractures and managing adverse events of bisphosphonates and denosumab, with approximately the same proportions (25–42%) reporting low or very low confidence and 17–36% did not respond. Increased education of nurses around bone health could help to increase confidence in dealing with these events.

This work also raises questions about the professional value that is attributed to specialist oncology nurses. Several common themes emerged regarding barriers to better awareness of the prevention of bone loss and risk factors for bone loss and to specialist care and treatment with a bone-targeted agent. These were related to lack of training or knowledge, lack of time for professional development or for patients and lack of funding for specialist nurses. The finding that a high proportion of participants did not answer the question about their role in patient care suggests that nurses’ roles and/or qualifications may not always be recognised or established as a resource for providing patient care. Interestingly, a relatively high proportion of participants selected ‘Other’ for the specific question about barriers to patients receiving bisphosphonates or denosumab for CTIBL, compared with the same response for other questions regarding barriers. This finding suggests that there are barriers to specialist oncology nurses’ management of CTIBL that remain to be identified.

The overall reported use of clinical treatment guidelines in the survey highlights a potential area for improvement. For example, in the management of patients with CTIBL, only 21.6%, 13.4% and 21.6% reported using international guidelines, country-specific guidelines and their institution guidelines, respectively. Increasing the uptake of clinical treatment guidelines may require the provision of sufficient time for specialist nurses to acquaint themselves with the guidelines and to refer to them while providing patient care. It is notable that institution guidelines were used more than country-specific guidelines. Previous research suggests that country-specific and international clinical guidelines may not be updated frequently enough to include information on newly approved drugs [22]. It is possible that institutions are better able than national or international guideline organisations to maintain their guidelines and to keep them relevant and useful to their staff. Therefore, support for local institutions to enable their staff to use their guidelines may help to improve the management of bone loss in patients receiving cancer treatment. Additionally, professional development of specialist nurses could include skills training for implementing guidelines in their work context.

### Impact of specialist nurses on the management of bone health

Research into the effectiveness of nursing and patient involvement in oncology has demonstrated the value of the interaction between nurses and patients in improving outcomes in cancer. Given that nurses have a greater opportunity than other healthcare professionals to build effective relationships with patients, patients may be better educated and empowered to report negative outcomes and treatment effects more frequently if specialist nurses’ awareness and knowledge of bone health issues in cancer were increased. It has been previously reported by nurses that a lack of understanding of bisphosphonates was the main barrier to providing patient education [17]. A literature review currently underway [8] may identify other specialist nursing activities that could be targeted in future educational programmes, which could be unified within the European Higher Education Area [27] and linked with the European Credit Transfer and Accumulation System.

### Limitations and strengths

The data provided by cross-sectional observational studies have inherent limitations. One possible limitation is sampling bias, in that the population of nurses who contributed to the survey may have had a particular interest in bone health and, considering that the survey was distributed via nursing societies, in their professional development. However, we consider the effect of such a bias to be a strength of this study, such that the suggestion for improved education in specialist oncology nurses is well grounded.

In addition, because the sample size was limited, this survey could not analyse possible differences in responses according to background or experience, nor could it analyse possible differences among countries, which may have guidelines that promote different management strategies (e.g. use of fracture T-scores or quality of life as indicators for treatment). Furthermore, because the data are from a single point in time, this survey cannot ascertain the impact of education on the approaches to managing bone health in cancer. Certainly, there are large differences in professional development strategies for nurses across Europe [1] and it would be helpful to determine the most effective strategies if specialist nurse training at all levels could be standardised through accreditation by organisations such as the European Accreditation Network for Educational Activities [15]. The participants were recruited through several different sources: a link was available through the EAUN website and it was advertised at the European Oncology Nurses Society Congress and through direct email from several nursing organisations. These organisations were chosen as they represent both oncology and urology nurses who are likely to treat patients with bone metastases and CTIBL regularly. There were no specific requirements for participants to provide evidence that they were specialist nurses before being able to complete the survey; however, because the survey was distributed through specialist nursing societies, we are confident that the responders were likely to be specialist nurses in oncology and urology fields. The second, third and fourth most common geographical locations of institutes were in countries where nurses were directly contacted by email (Ireland, Spain and Australia, respectively); therefore, it is likely that this direct contact led to increased responses. This does not, however, explain why nurses in Switzerland were so responsive. There was a high participation rate from some individual countries but the survey generated replies from a wide range of countries, a finding that we consider to be a strength of this study. The same could be said regarding therapeutic areas represented—although urological (among others) nursing societies were contacted, the nurses reported having a wide breadth of specialisations.

The data set captured all responses by including all participants who answered at least one question. This improved the analyses by maximising the number of responses and including the views of participants who were not able to answer all of the questions. For many questions, this resulted in high proportions of participants who did not answer. It cannot be assumed that those who did not respond would have given similar responses to those given by participants who did respond; therefore, the findings for each question may have been subject to response bias such that the differences in responses could have been either overestimated or underestimated [33]. It is beyond the capability of this survey to ascertain the source or effect of any such bias; however, because the survey questions did not include ‘do not know’ options, it is possible that participants who were unable to respond to a question for whatever reason (e.g. subject unfamiliarity or fatigue) chose to not answer. Therefore, the findings probably indicate the maximum levels of awareness and knowledge possible in this study population. Additionally, the high proportions of participants not responding to some questions support the need for improved education on bone health for specialised oncology nurses. In designing future surveys, a requirement to complete all parts of the survey, alongside an ‘I do not know’ option would eliminate the problem of missing data; however, it is likely that the number of participants who complete the survey would decrease.

This work did not include an internal consistency analysis, but there are some findings that suggest that responses to questions overall were consistent with each other. For example, the proportions of participants who were aware of and who used guidelines for the management of patients with bone metastases were similarly low and, in another part of the survey, participants gave inadequacy of guidelines as one of the barriers to the management of bone health.

## Conclusion

Specialist oncology nurses play an important supporting role in the prevention of bone complications, diagnosis and treatment of patients with bone metastases and those at risk of CTIBL; however, nurses may not always be aware of how they can support patients’ bone health. As part of a multidisciplinary approach, specialist nurses have key opportunities while performing baseline assessments and ongoing monitoring to inform patients about their disease and treatment and to empower them to report symptoms or adverse events. Improvements in specialist oncology nursing practice can benefit patients not only through better management of their bone health but also by improving their quality of life and survival. There is a need for considerable improvement in the education and training of specialist nurses, and it is likely that practical educational programmes and training courses in bone health would be in high demand if they were available and fully accredited for use in more than one country. Given that nursing is subject to increasing budget restraints in many countries, training may also be appropriate for non-specialist nurses to allow proper assessment and referral of patients with cancer who are at risk of bone complications. Research into the requirements of and outcomes from improved education for specialist oncology nurses should be continued.

## Electronic supplementary material

ESM 1 (DOCX 41.8 kb)
