# Supplementary material for: Management of bone health in patients with cancer: a survey of specialist nurses
Source: Support Care Cancer. 2019 Jun 15;28(3):1151–62. doi: 10.1007/s00520-019-04858-2 (PMC6989658; doi:10.1007/s00520-019-04858-2)
Supplement: Supplementary file 1 — (DOCX 41.8 kb) [file 520_2019_4858_MOESM1_ESM.docx]

# Online resources for:

# Management of bone health in patients with cancer: a survey of specialist nurses

Lawrence Drudge-Coates,^1^ Erik van Muilekom,^2^ Julio C de la Torre-Montero,^3^ Kay Leonard,^4^ Marsha van Oostwaard,^5^ Daniela Niepel,^6^ Bente Thoft Jensen^7^

^1^Department of Urology, King’s College Hospital NHS Foundation Trust, London, United Kingdom; ^2^Antoni van Leeuwenhoek, Netherlands Cancer Institute, Amsterdam, Netherlands; ^3^Comillas Pontifical University, San Juan de Dios School of Nursing and Physical Therapy, Madrid, Spain; ^4^St Luke's Radiation Oncology Centre at St James's Hospital, Dublin, Ireland; ^5^Máxima Medisch Centrum, Eindhoven/Veldhoven, Netherlands; ^6^Amgen (Europe) GmbH, Rotkreuz, Switzerland; ^7^Aarhus University Hospital and Centre of Research in Rehabilitation, Aarhus University, Aarhus, Denmark

**Table of contents**

| **Title** | **Page** |
| --- | --- |
| Online Resource 1. Steering committee members | 2 |
| Online Resource 2. Oncology nurse survey – bone health (English version) | 3 |

**Online Resource 1. Steering committee members.**

Bente Thoft Jensen (Aarhus University Hospital and Centre of Research in Rehabilitation, Aarhus University, Aarhus, Denmark)

Kay Leonard (St Luke’s Radiation Oncology Centre at St James’s Hospital, Dublin, Ireland)

Julio C de la Torre-Montero (Comillas Pontifical University, San Juan de Dios School of Nursing and Physical Therapy, Madrid, Spain; Medical Oncology Department, Hospital Clínico Universitario San Carlos, Instituto de Investigación [IdISSC], Madrid, Spain)

**Online Resource 2. Oncology nurse survey – bone health (English version)**

| **Q1. How many years of experience do you have in oncology?** |
| --- |
| < 1 |
| 1 to < 4 |
| 4 to < 8 years |
| 8 to < 12 years |
| 12 to < 15 years |
| ≥ 15 years |
| **Q2. Which therapeutic area(s) do you specialise in (please tick all that apply)?** |
| Urology |
| Breast cancer |
| Radiotherapy |
| Medical oncology |
| Cancer treatment-induced bone loss (CTIBL = osteoporosis) |
| Palliative care |
| Geriatric care |
| Paediatric care |
| Bone health |
| Orthopaedics |
| Surgery |
| No specialism |
| If you do not currently work in these fields, thank you for your interest in the survey. The remaining questions do not apply |
| Other (please specify) |
| **Q3. How did you become a specialist nurse (please tick all that apply)?** |
| Specialist programme/education certification (please specify) |
| Educational background |
| > 2 years of experience in clinical practice |
| > 5 years of experience in clinical practice |
| Not applicable |
| Other (please specify) |
| **Q4. At your institution, what's the definition of a nurse specialist (please tick all that apply)?** |
| Specialist programme/education certification (please specify) |
| Educational background |
| > 2 years of experience in clinical practice |
| > 5 years of experience in clinical practice |
| Not applicable: no availability of a specialised educational programme in uro-oncology nursing |
| Not applicable: no national recognition of the field of advanced specialist nursing |
| Other (please specify) |
| **Q5. What type(s) of patient(s) do you work with (please tick all that apply)?** |
| Adults |
| Adolescents |
| Children |
| **Q6. Which setting do you work in (please tick all that apply)?** |
| Outpatient clinics |
| Inpatient wards |
| Community care |
| Other (please specify) |
| **Q7. Which type of institution do you work in (please tick all that apply)?** |
| Private |
| Public |
| Mixed |
| General hospital |
| Comprehensive cancer centre |
| Other (please specify) |
| **Q8. In which country is your institution based?** |
| Indicate country |
| **Q9. How confident do you feel about identifying patients at risk of CTIBL (cancer treatment-induced bone loss) in your everyday practice (1 = not confident, 4 = extremely confident)?** |
| 1 |
| 2 |
| 3 |
| 4 |
| I do not assess or manage patients with CTIBL |
| **Q10. How confident do you feel about identifying patients at risk of fracture in your everyday practice (1 = not confident, 4 = extremely confident)?** |
| 1 |
| 2 |
| 3 |
| 4 |
| **Q11. How many of the risk factors for hip fracture listed below were you aware of (please tick all that apply)?** |
| Age older than 65 years |
| Androgen-deprivation therapy |
| Aromatase inhibitor therapy |
| Ovarian suppression therapy |
| Family history of osteoporosis or hip fracture |
| Female gender |
| History of alcohol consumption |
| History of smoking |
| Long-term glucocorticoid therapy |
| Low body mass index |
| Low bone mineral density |
| Prior fragility fracture |
| Rheumatoid arthritis |
| I was not aware of any risk factors |
| Other (please specify) |
| **Q12. Which of these measures for the prevention of bone loss are you aware of (please tick all that apply)?** |
| Adequate calcium intake |
| Fall prevention strategies |
| Reducing alcohol intake |
| Stopping smoking |
| Treatment with bone-targeted agents |
| Vitamin D supplements |
| Weight-bearing exercise |
| I was not aware of any preventive measures |
| **Q13. At your institution, what are the main barriers to better awareness of the risk factors for bone loss (please tick all that apply)?** |
| Lack of funding for other healthcare professionals |
| Lack of funding for specialist nurses |
| Lack of time for professional development |
| Lack of knowledge of the evidence of effective interventions |
| Lack of training |
| Inadequacy of international clinical practice guidelines |
| Inadequacy of government/country-specific guidelines |
| There are no barriers |
| Other (please specify) |
| **Q14. At your institution, what are the main barriers to better awareness of preventive measures for bone loss (please tick all that apply)?** |
| Lack of funding for other healthcare professionals |
| Lack of funding for specialist nurses |
| Lack of time for professional development |
| Lack of knowledge of the evidence of effective interventions |
| Lack of training |
| Inadequacy of international clinical practice guidelines |
| Inadequacy of government/country-specific guidelines |
| There are no barriers |
| Other (please specify) |
| **Q15. Do you agree or disagree with the statement “I fully understand the potential complications that may result from inadequate management of patients with CTIBL” (1 = completely disagree, 4 = completely agree)?** |
| 1 |
| 2 |
| 3 |
| 4 |
| **Q16. Are you aware of guidelines for the management of patients with CTIBL?** |
| No |
| Yes (if yes, please tick all that apply): |
| International guidelines (e.g. the European Society for Medical Oncology [ESMO]) |
| Government/country-specific guidelines (e.g. National Institute for Health and Care Excellence [NICE]) |
| Institution’s own guidelines |
| Other (please specify) |
| **Q17. At your institution, do you use any of the following guidelines when making decisions on the management of patients with CTIBL (please tick all that apply)?** |
| International guidelines (e.g. ESMO) |
| Government/country-specific guidelines (e.g. NICE) |
| Institution’s own guidelines |
| Other (please specify) |
| **Q18. At your institution, which of these areas of the management of CTIBL do nurses have a role in (please tick all that apply)?** |
| Educating patients on fracture prevention |
| Educating patients on their fracture risk |
| Educating patients on the importance of adherence to treatment for bone loss |
| Educating patients on the potential adverse effects of treatment |
| Identifying patients at high risk of fracture |
| Initiating treatment for CTIBL |
| Screening patients for low bone mineral density |
| Nurses do not have a role in the treatment of CTIBL |
| Other (please specify) |
| **Q19. At your institution, how is fracture risk assessed in patients with cancer (please tick all that apply)?** |
| Identifying fracture risk fractures (e.g. age, gender, history of fragility fracture) |
| Screening patients for low bone mineral density (e.g. using dual energy X-ray absorptiometry [DXA] scans) |
| Using the FRAX® tool |
| Regular height measurement |
| We do not assess fracture risk |
| Other (please specify) |
| **Q20. At your institution, when do patients initiate treatment with bone-targeted agents such as bisphosphonates or denosumab (please tick all that apply)?** |
| When patients are prescribed an aromatase inhibitor, ovarian suppression or androgen-deprivation therapy |
| When patients have multiple risk factors (e.g. age, history of fragility fracture) for fracture (regardless of T-score) |
| When patients have a T-score < −2.0 |
| When patients have a T-score < −2.0 and additional risk factors for fracture |
| We do not use bone-targeted agents for CTIBL |
| Other (please specify) |
| **Q21. At your institution, what are the barriers to patients receiving bone-targeted agents for CTIBL (please tick all that apply)?** |
| Availability of bone-targeted agents |
| Budget constraints |
| International guidelines |
| Government/country-specific guidelines |
| Institution guidelines |
| Lack of awareness of bone-targeted agents for CTIBL |
| Lack of training in the use of bone-targeted agents |
| Lack of knowledge of pain assessment |
| Lack of knowledge of nutritional screening |
| Lack of knowledge on how to identify patients who could benefit from treatment |
| Other (please specify) |
| **Q22. How confident do you feel about managing patients with bone metastases in your everyday practice (1 = not confident, 4 = extremely confident)?** |
| 1 |
| 2 |
| 3 |
| 4 |
| **Q23. Do you agree or disagree with the statement “I fully understand the potential complications that may result from inadequate management of bone metastases and associated complications” (1 = completely disagree, 4 = completely agree)?** |
| **1** |
| 2 |
| 3 |
| 4 |
| **Q24. How confident do you feel about your knowledge on the prevention of skeletal-related events (SREs; e.g. pathologic fracture, surgery to bone, radiation to bone and spinal cord compression) and bone complications (e.g. bone pain) in your everyday practice (1 = not confident, 4 = extremely confident)?** |
| 1 |
| 2 |
| 3 |
| 4 |
| **Q25. How confident do you feel about preventing and managing side effects associated with bone-targeted agents in your everyday practice (1 = not confident, 4 = extremely confident)?** |
| 1 |
| 2 |
| 3 |
| 4 |
| **Q26. Are you aware of guidelines for the management of patients with bone metastases (please tick all that apply)?** |
| No |
| Yes (if yes, please tick all that apply): |
| International guidelines (e.g. ESMO) |
| Government/country-specific guidelines (e.g. NICE) |
| Institution’s own guidelines |
| I am not aware of any guidelines |
| Other (please specify) |
| **Q27. At your institution, who is mainly responsible for managing bone health?** |
| Physician |
| Specialist nurse |
| Nurse |
| Other (please specify) |
| **Q28. At your institution, do you use any of the following guidelines when making decisions on the management of patients with bone metastases (please tick all that apply)?** |
| International guidelines (e.g. ESMO) |
| Government/country-specific guidelines (e.g. NICE) |
| Institution’s own guidelines |
| Other (please specify) |
| **Q29. At your institution, are nurses part of a multidisciplinary team that focus on pain management, palliative care or bone metastases?** |
| Yes |
| No |
| Sometimes |
| **Q30. At your institution, what are the barriers to working as a multidisciplinary team (please tick all that apply)?** |
| Lack of funding |
| Lack of specialist healthcare professionals |
| Lack of training |
| No formal requirement for interdisciplinary working |
| Physicians are solely responsible for patients’ care |
| There are no barriers |
| Other (please specify) |
| **Q31. At your institution, are there any specialist nurses who are involved in the care of cancer patients?** |
| Yes (please specify their role in patient care) |
| No |
| **Q32. At your institution, what are the barriers to every patient receiving care from a specialist nurse (please tick all that apply)?** |
| Lack of funding |
| Lack of specialist nurses |
| Lack of training |
| No formal requirement for specialist nurse provision |
| Physicians are solely responsible for patients’ care |
| There are no barriers |
| Other (please specify) |
| **Q33. Which of these roles do you play in patient care (please tick all that apply)?** |
| Prescription of medication |
| Administering treatment/diagnostic interventions |
| Monitoring disease progression |
| Pain management/palliative care |
| Undertaking diagnostic tests (e.g. biopsy, imaging [e.g. dual energy X-ray absorptiometry]) |
| Patient advocacy |
| Education of nurses |
| Providing information for patients on the adverse effects of treatment |
| Providing information for patients on treatment options |
| Providing psychosocial support for patients |
| Other (please specify) |
| **Q34. At your institution, do you think that nurses are able to meet the following patients’ needs (please tick all that apply)?** |
| Information needs |
| Psychological support |
| Symptom management |
| Treatment choice |
| **Q35. At your institution, what are the main barriers to nurses optimally managing the needs of patients with bone metastases (please tick all that apply)?** |
| Lack of appropriate training |
| Lack of authority to make key decisions on patient care |
| Lack of time to spend with patients |
| Need to meet alternative government targets |
| The focus is on care for patients with early-stage cancer rather than advanced cancer |
| There are no barriers |
| Other (please specify) |
| **Q36. At your institution, which of these measures are regularly used to prevent adverse events in patients receiving bone-targeted agents (please tick all that apply)?** |
| Monitoring renal function (serum creatinine) |
| Adequate hydration |
| Monitoring calcium levels |
| Recommending vitamin D and calcium |
| Completing dental work before starting treatment |
| Proactive dental surveillance |
| Other (please specify) |
| **Q37. If all of the measures in question 36 are not regularly used, what are the main barriers to their use (please tick all that apply)?** |
| Budget constraints |
| Inadequacy of international clinical practice guidelines |
| Inadequacy of government/country-specific guidelines |
| There are no barriers |
| Other (please specify) |
| **Q38. At your institution, when do patients usually initiate treatment with bone-targeted agents for the prevention of skeletal-related events (SREs; please tick all that apply)?** |
| At diagnosis of advanced cancer |
| At diagnosis of castration-resistant prostate cancer |
| At diagnosis of bone metastases |
| At diagnosis of an SRE |
| Following multiple SREs |
| Patients are not treated with bone-targeted agents |
| Other (please specify) |
| **Q39. At your institution, what are the barriers to prescribing bone-targeted agents to patients at an earlier stage in their disease than you do currently (please tick all that apply)?** |
| Availability of bone-targeted agents |
| Budget constraints |
| Inadequacy of international clinical practice guidelines |
| Inadequacy of government/country-specific guidelines |
| Lack of predictive factors that identify which patients would benefit from early treatment |
| There are no barriers |
| Other (please specify) |

CTIBL, cancer treatment-induced bone loss.
